# Development of Biocompatible HA Hydrogels Embedded with a New Synthetic Peptide Promoting Cellular Migration for Advanced Wound Care Management

**DOI:** 10.1002/advs.201800852

**Published:** 2018-09-21

**Authors:** Sun Young Wang, Hyosuk Kim, Gijung Kwak, Hong Yeol Yoon, Sung Duk Jo, Ji Eun Lee, Daeho Cho, Ick Chan Kwon, Sun Hwa Kim

**Affiliations:** ^1^ KU‐KIST Graduate School of Converging Science and Technology Korea University Seoul 02841 Republic of Korea; ^2^ Center for Theragnosis Biomedical Research Institute Korea Institute of Science and Technology (KIST) Seoul 02792 Republic of Korea; ^3^ Nano‐Bio Resources Center Sookmyung Women's University Seoul 04310 Republic of Korea

**Keywords:** bioorthogonal copper free click chemistry, peptide‐grafted hyaluronic acid hydrogels, would healing peptides, wound repair

## Abstract

In the past few years, there have been many efforts underway to develop effective wound healing treatments for traumatic injuries. In particular, wound‐healing peptides (WHPs) and peptide‐grafted dressings hold great promise for novel therapeutic strategies for wound management. This study reports a topical formulation of a new synthetic WHP (REGRT, REG) embedded in a hyaluronic acid (HA)‐based hydrogel dressing for the enhancement of acute excisional wound repair. The copper‐free click chemistry is utilized to form biocompatible HA hydrogels by cross‐linking dibenzocyclooctyl‐functionalized HA with 4‐arm poly(ethylene glycol) (PEG) azide. The HA hydrogels are grafted with the REG peptide, a functional derivative of erythroid differentiation regulator1, displaying potent cell motility‐stimulating ability, thus sustainably releasing physiologically active peptides for a prolonged period. Combined with the traditional wound healing benefits of HA, the HA hydrogel embedded REG (REG‐HAgel) accelerates re‐epithelialization in skin wound healing, particularly by promoting migration of fibroblasts, keratinocytes, and endothelial cells. REG‐HAgels improve not only rate, but quality of wound healing with higher collagen deposition and more microvascular formation while being nontoxic. The peptide‐grafted HA hydrogel system can be considered as a promising new wound dressing formulation strategy for the treatment of different types of wounds with combinations of various natural and synthetic WHPs.

## 1 Introduction

Peptide‐based therapeutic agents offer enormous potential for addressing unmet medical needs due to their high specificity, efficacy, and safety.^1^ In the last decade, considerable efforts have been devoted to find bioactive peptides possessing wound healing properties for developing effective wound care treatments. Most current biomedical approaches to advanced wound care aim at boosting normal healing events using novel therapeutic peptides originated from either natural or synthetic sources, particularly formulated as functional wound dressings to accelerate reestablishment of the skin tissue.^2^

In this study, a novel synthetic peptide REGRT (AES16‐2M, denoted as REG), inspired by erythroid differentiation regulator1 (Erdr1) known as a stress‐related survival factor, was used to prepare durable advanced wound dressings for the topical treatments in excisional wound healing. The REG peptide was found to stimulate wound‐healing via promoting cell migration and tissue invasion in fibroblasts, keratinocytes, and vascular endothelial cells. The peptide contributed to the cell migratory ability especially through the up‐regulation of matrix metalloproteinases (MMPs) via the Rac1‐ERK signaling pathway. Although peptides are relatively easy to be delivered across biological barriers than larger proteins due to the smaller size, they still face significant challenges in the formulary aspects due to low permeation, poor retention, and proteolytic instability.[[qv: 1a]] Topical drug formulations can supply physiologically active peptides over a sustained period, increasing the wound residence time of drugs to maintain their local concentrations within therapeutic ranges.^3^ In particular, hydrogel dressing formulations possess several favorable properties for wound healing, such as maintaining a moist wound environment, providing a barrier against microorganisms, and allowing gaseous exchange, and removing wound exudates.^4^ Thus, hydrogel‐based wound dressings containing therapeutic peptides can be extremely useful for advanced wound healing and skin regeneration.

**Figure 1:**
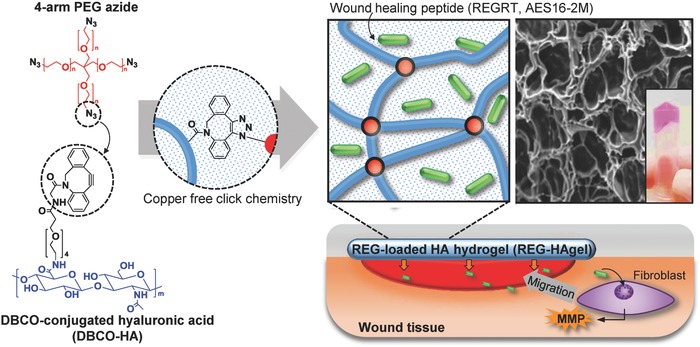
Schematic illustration of hyaluronic acid hydrogel (HAgel) preparation through copper‐free click chemistry, encapsulation of REG peptides, and its application in wound repair through activating cellular migration. Cross‐sectional SEM image and a photograph of HAgel represent formation of cross‐linked hydrogel. For the photograph of HAgel, HAgel was shortly submerged in DMEM culture medium to give the pink color.

To design a potential topical delivery system for the REG peptide, a natural polymeric material, hyaluronic acid (HA), was employed in the formulation of REG‐grafted hydrogel wound dressings (REG‐HAgel) (**Figure** **1**). HA, an essential component of extra cellular matrix, is well known for its hydrophilic and water retaining properties^5^ and considered to be one of the key components of the wound healing process, modulating inflammation, cellular migration, and angiogenesis.^6^ With the aforementioned physical and physiological properties, HA‐based hydrogel wound dressings can serve a dual function as active drug carriers and wound healing agents. Traditional HA‐based hydrogels have utilized various cross‐linking agents such as glutaraldehyde, divinyl sulfone, 1,2,3,4‐diepoxybutane, and carbodiimide, which have cellular toxicity and genotoxicity.^7^ Although these chemical agent‐based cross‐linking systems can be used in large scale production with low cost, they have potential side effects after treatment. In the case of HA‐based hydrogels cross‐linked using copper‐catalyzed click chemistry, they showed rapid reaction time and high chemoselectivity at physiological pH and temperature. However, the copper‐catalysts should be purified from the final products, since copper generates reactive oxygen species that cause oxidative DNA damage.^8^ As an alternative approach for preparing HAgels, copper‐free click chemistry based on cyclooctyne‐azide cycloaddition reaction requires no catalysts and also shows rapid reaction time with high chemoselectivity at physiological pH and temperature and is proven to show good biocompatibility, which is suitable for biomedical applications.^9^

Here, a new engineered HA hydrogel, REG‐Hagel, was synthesized through strain‐promoted cyclooctyne‐azide cycloaddition reaction in aqueous solutions containing the REG peptides. This copper‐free click chemistry allows spontaneous conjugation in physiological temperature and pH ranges and is proven to show good biocompatibility.[[qv: 9b]] First, high‐molecular weight HA‐dibenzocyclooctyl (DBCO‐HA) derivatives were prepared through 1‐ethyl‐3‐(3‐dimethylaminopropyl) carbodiimide (EDC)/N‐hydroxysuccinimide (NHS) coupling reaction, and HA hydrogels were cross‐linked with 4‐arm poly(ethylene glycol) (PEG) azide through click chemistry. During the process of hydrogel fabrication, the REG peptides were encapsulated through simply mixing with DBCO‐HA solution. The physicomechanical properties and biocompatibility of REG‐HAgel were characterized, and its therapeutic potential in vitro and in vivo was investigated in neonatal human dermal fibroblast (nHDF) cell line and murine excisional acute wound model, respectively.

## 2 Results and Discussion

### 2.1 Promoting Cellular Migration with REG Peptides

**Figure 2:**
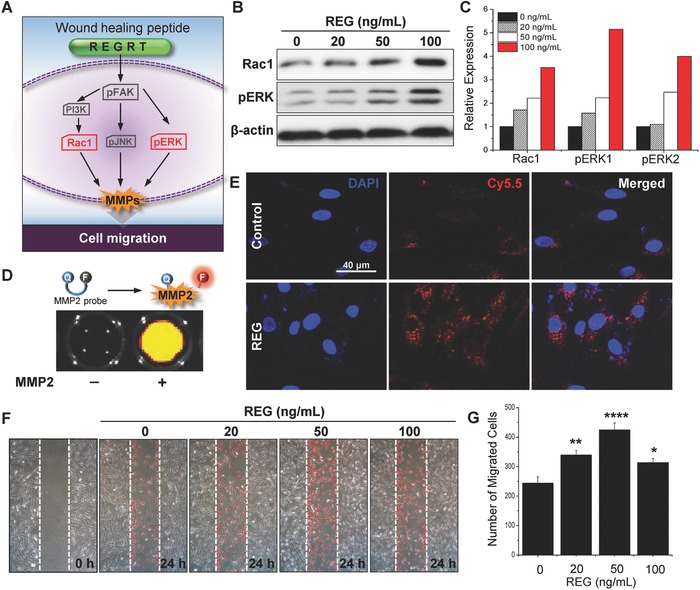
In vitro therapeutic effect of REG peptide. A) Schematic illustration of signaling pathways affected by REG peptide. B) Western blot analysis of Rac1, pERK1/2, and β‐actin with respect to different concentrations of REG and C) quantification of expression levels. D) Mechanism of MMP2‐activatable probe for visualization of MMP2 activity and fluorescence image representing recovery of fluorescence in the presence of MMP2. E) Fluorescence images showing MMP2 activity of cells treated with REG. F) Wound scratch migration assay with different concentrations of REG peptide and G) quantification of migrated cells. *n* = 4; *p < 0.05, **p < 0.01, ****p < 0.0001 versus saline‐treated cells.

REG (AES16‐2M) is a new synthetic wound healing peptide derived from the functional region of Erdr1, a highly conserved autocrine factor regulating stress‐related responses.^10^ Immunogenicity is strongly correlated with the size of substances and generally macromolecules with a molecular mass less than 5000 Da are known as poor immunogens.^11^ Thus, REG consisting of only 5 amino acids (REGRT, *M*_w_ 617 Da) has no risk of antibody production. In addition, the REG peptide sharing three amino acid sequence GRT with Erdr1 displays a potent cell motility‐stimulating ability in various cell types including fibroblasts, keratinocytes, and vascular endothelial cells (**Figure** **2**; Figures S1 and S2, Supporting Information). In wound healing and regeneration, collective cell migration plays a pivotal role during proliferative phase. In particular, fibroblasts known as leader cells driving the collective migration also support re‐epithelialization by producing collagen, a major component of extracellular matrix (ECM), and stimulate capillary growth and granulation.^12^

One way of promoting fibroblast migration is activating focal adhesion kinase (FAK) signaling pathways (Figure 2A). Interaction between FAK and PI3 kinase induces effector Rac, which regulates cortical actin and lamellipodia in cell migration.^13^ Extracellular signal‐regulated kinase (ERK)/mitogen‐activated protein (MAP) kinase is another downstream signaling pathway of FAK, as active ERK increases actin–myosin contraction of cells.^14^ In vitro studies using the novel synthetic peptide REG clarified the molecular signaling mechanisms of cell migration especially in nHDF. As show in Figure 2B,C, the protein levels of Rac1 and pERK were increased in direct proportion to the concentration of REG. With the peptide concentration of 100 ng mL^−1^, the protein expression levels of Rac1, pERK1, and pERK2 were fourfold, sixfold, and fivefold, respectively, higher than those of the control group.

Activation of both Rac1 and ERKs can up‐regulate MMPs that promote MMP‐dependent cell migration and invasion through ECM degradation.^14^ In Figure 2D,E, the dynamic MMP2 activity of the REG‐treated cells was visualized using MMP2‐activatable probe technology developed in our previous research.^15^ The MMP2 probe composed of fluorogenic peptide‐Cy5.5 dye (PLGLR‐Cy5.5) and near‐infrared (NIR) dark‐quencher (BHQ‐3) was synthesized with standard solid‐phase Fmoc peptide chemistry analyzed by reverse‐phase high‐performance liquid chromatography (HPLC) (Figure S3, Supporting Information) and used after purification. When the probe is exposed to MMP2, enzymes cleave the fluorogenic peptide and separate the quencher, allowing its fluorescence to be detected. The confocal fluorescence imaging using MMP2 probe clearly showed that active MMP2 was highly expressed in nHDFs after 15 h of REG stimulation. To assess cell migratory activity induced by REG, in vitro wound scratch assay was performed in different cell lines including fibroblasts, keratinocytes, and vascular endothelial cells (Figure 2F,G; Figures S1 and S2, Supporting Information). The REG peptides indeed promoted the migration of different cell types, especially with a dramatic effect on the migration of fibroblasts acting as leader cells. As the REG concentration increased, the number of migrated cells gradually increased until the concentration reached 50 ng mL^−1^ and then decreased thereafter, likely due to some saturation effect attributed to high concentration of bioactive peptides. Although excess amounts of active Rac1 and ERK were observed at REG concentrations above 100 ng mL^−1^, the rate of cell migration mediated via the Rac1‐ERK signaling pathway was rather reduced. This can be attributed to the negative feedback regulation of ERK signaling in normal cells.^16^ Taken together, these results demonstrated that the REG peptide could accelerate MMP‐induced cell migration via activating Rac1‐ERK signaling pathways.

### 2.2 Synthesis and Characterization of HA Hydrogels Cross‐Linked via Copper‐Free Click Chemistry

**Figure 3:**
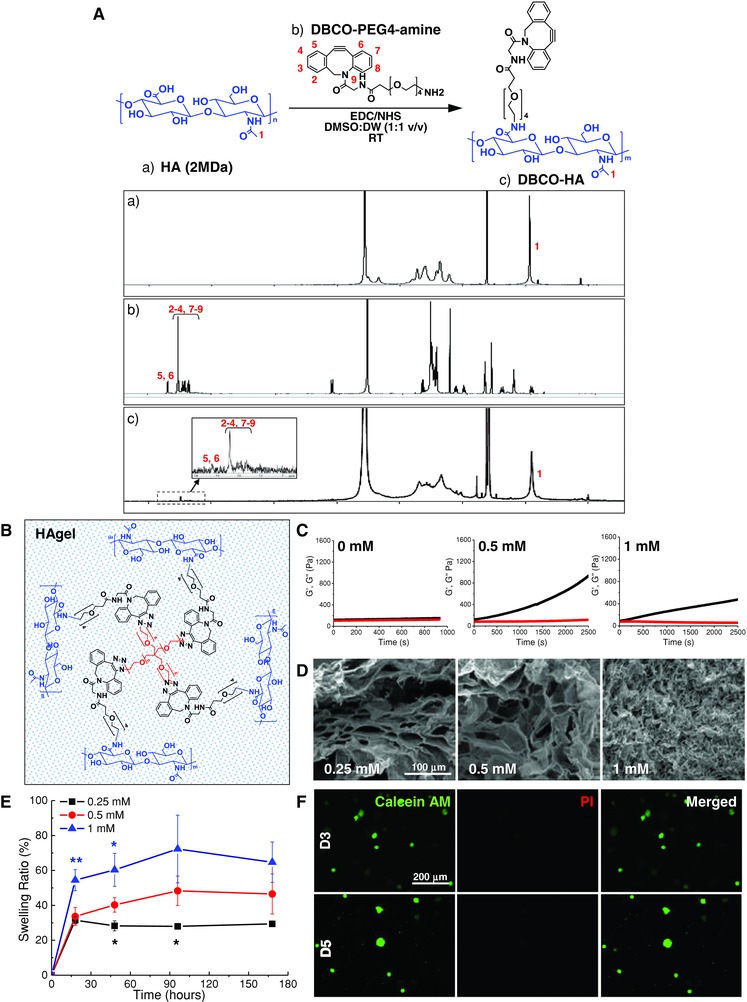
Characterization of HAgel. A) Chemical composition analysis with ^1^H‐NMR confirming the synthesis of DBCO‐HA. B) Schematic diagram of HAgel formation. C) Elastic modulus with black dots representing storage modulus (*G*′) and red dots representing loss modulus (*G*″). D) Scanning electron microscope (SEM) images showing porous structure of Hagel. E) Swelling ratio of HAgel. *n* = 6; *p < 0.05, **p < 0.01 versus 0.5 × 10^−3^ m cross‐linker. F) Live‐dead cell staining of cells encapsulated in HAgel.

As a topical delivery system for sustained release of REG at the wound site, in situ formed biocompatible HA‐based hydrogels were synthesized using a copper‐free click chemistry reaction (Figure 1). First, DBCO‐PEG_4_‐HA (DBCO‐HA) derivatives were prepared by conjugating DBCO‐PEG_4_‐amine and HA using EDC and NHS reagents. As shown in **Figure** **3**A, the chemical composition of DBCO‐HA was confirmed by proton nuclear magnetic resonance (^1^H‐NMR) analysis of the individual compounds (HA, DBCO‐PEG_4_‐amine, and DBCO‐HA). The peaks at 2 ppm, labeled as 1, and 7.3–7.8 ppm, labeled as 2 through 9, indicated the presence of N‐acetyl glucose amine protons of HA and DBCO protons of DBCO‐PEG_4_‐amine, respectively. This result suggested that the carboxyl group in HA was successfully modified with DBCO by the EDC/NHS‐mediated coupling reaction. Here, 4‐arm PEG azide was used as a bioorthogonal cross‐linker due to its well‐defined symmetric structure, high hydrophilicity, and commercial availability.^17^ The synthesized DBCO‐HA was cross‐linked with 4‐arm PEG azide under physiological conditions to prepare HAgels (Figure 3B). In the attenuated total reflectance (ATR) Fourier transform infrared (FT‐IR) spectrum of HA‐DBCO, the peak of aromatic C=C bending was observed at 1800–1750 cm^−1^ (Figure S4, Supporting Information). After mixing 0.5 × 10^−3^ m of 4‐arm‐PEG‐azide with 20 mg mL^−1^ of HA‐DBCO, C—N stretching was found at 1260–1240 cm^−1^. As expected, the azide stretching peak at 2100–2120 cm^−1^ was rarely observed in the spectrum of HAgel, implying that HA‐DBCO was successfully conjugated with 4‐arm‐PEG‐azide via copper‐free click chemistry.

The HAgels formed with different concentrations of 4‐arm PEG azide cross‐linker were characterized by oscillatory rheology, scanning electron microscopy (SEM), and swelling ratio. The elastic modulus was determined by measuring storage modulus (*G*′) and loss modulus (*G*″) at room temperature (Figure 3C). DBCO‐HA began to gelate at the cross‐linker concentration of 0.25 × 10^−3^ m (data not shown). The elastic modulus of HAgels increased corresponding to the cross‐linker concentration and showed a peak at 0.5 × 10^−3^ m. The HAgels with 1 × 10^−3^ m 4‐arm PEG azide exhibited rather lower elastic modulus. Cross‐sectional microstructure morphologies of HAgels were visualized using scanning electron microscopy (Figure 3D). Overall, in the presence of varying concentrations of cross‐linker, hydrogels exhibited a highly interconnected porous structure with different pore sizes. While the pore sizes of the hydrogels with 0.25 and 0.5 × 10^−3^ m 4‐arm PEG azide were maintained at about 10–50 µm, the one with 1 × 10^−3^ m cross‐linker exhibited extremely dense structure with pores in the nanometer size range. The swelling behavior of HAgels was evaluated by measuring the weights of hydrogels in the initial dried state and swollen state after incubating in saline at pH 7.4 over time (Figure 3E). The swelling ratio was measured until no further weight increase was observed. The equilibrium swelling ratio of HAgels gradually increased with increasing concentration of 4‐arm PEG azide. In the case of HAgels with 1 × 10^−3^ m 4‐arm PEG azide, however, the swelling ratio decreased rather after 95 h, and the hydrogels started to rapidly dissolve in aqueous solutions. The gel mass completely disappeared after 240 h, indicating poor cross‐linking level of the gels. Based on the data from physical characterization of HAgels, the hydrogel formulation prepared at 0.5 × 10^−3^ m 4‐arm PEG azide concentration was chosen and further utilized to prepare peptide‐grafted HAgel. In addition, in vitro biocompatibility assessment revealed that our bioorthogonal cross‐linking method had no harmful effects of HAgels on nHDFs (Figure 3F).

### 2.3 Fabrication of REG‐HAgels and Controlled Release of Active Components from the Gels

**Figure 4:**
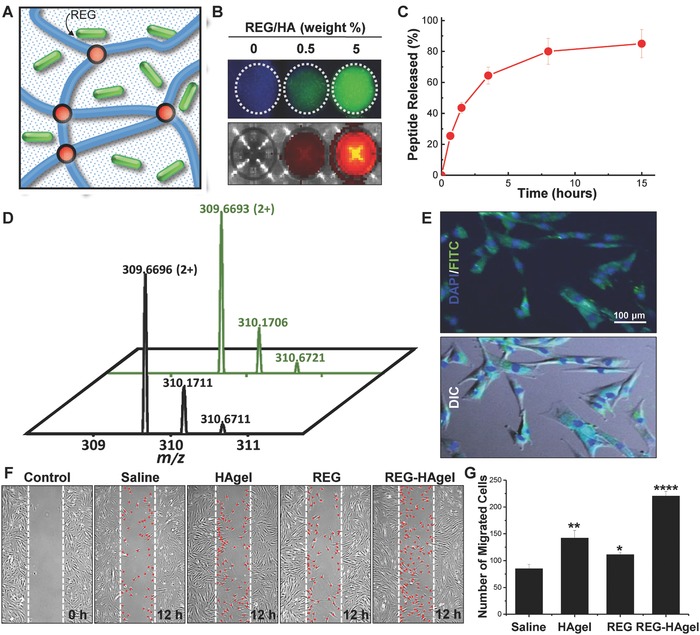
Characterization of REG‐HAgel in vitro. A) Illustration of REG peptide encapsulation. B) Fluorescence image of FITC‐labeled REG released from HAgel in saline solution. Gels were incubated for 4 h. C) Release profile of FITC‐labeled REG from HAgel up to 15 h of incubation. *n* = 3. D) Mass spectrometry analysis with green line indicating REG peptide released from HAgel and black line indicating REG peptide dissolved in deionized water. E) Fluorescence image of cellular uptake of FITC‐labeled REG. Images were taken 4 h after treating with FITC‐labeled REG‐loaded HAgel. F) Wound scratch assay with saline, HAgel, REG, and REG‐HAgel treatments and G) quantification of migrated cells. *n* = 4; *p < 0.05, **p < 0.01, ****p < 0.0001 versus saline‐treated cells.

REG‐HAgels were prepared by physically embedding the REG peptides within HAgels via adding the peptides during the fabrication process of hydrogels (**Figure** **4**A). To examine the peptide‐loading capacity and release kinetics of the hydrogels, we used fluorescein isothiocyanate (FITC)‐labeled REG peptide because we can facilely and robustly monitor the behavior of payloads as a function of time and hydrogel composition.^18^ REG‐HAgels containing different concentrations of FITC‐labeled REG were observed under UV lamp, and the free peptides released from the gels into the saline buffer were visualized with IVIS II Lumina (Figure 4B). The fluorescence intensity of REG‐HAgels was clearly increased with increasing peptide feed ratios, showing successful entrapment of the peptides within the hydrogel network. As expected, the physically entrapped peptides were completely released from the hydrogel within a few days. As shown in the release profile of the peptide (Figure 4C), REG‐HAgels exhibited typical diffusion controlled release with a relatively small initial burst followed by a slow sustained release. To investigate whether the peptide entrapment process influenced the physical stability and physiological activity of REG, mass spectrometry and wound scratch migration assay were additionally performed with the free REG peptides released from REG‐HAgels. As shown in Figure 4D, the free peptide released from REG‐HAgels exhibited exactly the same mass value (*M*_w_ 617.3241 Da) as the REG peptide dissolved in deionized water (*M*_w_ 617.3247 Da), corresponding to the theoretical mass of 617.3246 Da (REGRT). It supported that the synthetic peptide fragments maintained intact during the entrapment and release processes.

In addition, the REG peptides released from REG‐HAgels could be effectively delivered into nHDF cells (Figure 4E) and showed improved cell migratory activity as compared to the cell‐intrinsic migratory activity (Figure 4F,G). Besides its role as a peptide carrier, HAgels can offer additional benefits to wound healing. In particular, the interaction between HA and its specific receptor CD44 results in the pathological appearance of fibrosis.^19^ High MW‐HA (>500 kDa) is associated with anti‐inflammatory effects and increases the level of collagen III in dermal fibroblasts, while low MW‐HA (<500 kDa) activates AP‐1 and NFκB signaling cascades that improve fibroblast migration and proliferation.^20^ HAgels alone showed increased level of nHDF migration compared with the saline treatment, which can be attributed to the cleavage of HAgels into small HA fragments activating nHDF migration. Meanwhile, REG‐HAgels achieved maximum cell migration more than twofold higher than REG or HAgels, indicating the peptide and HA acted synergistically to improve migration of fibroblasts. Taken together, these in vitro results supported that REG was successfully encapsulated into HAgels and sustainably released as its physiologically active form.

**Figure 5:**
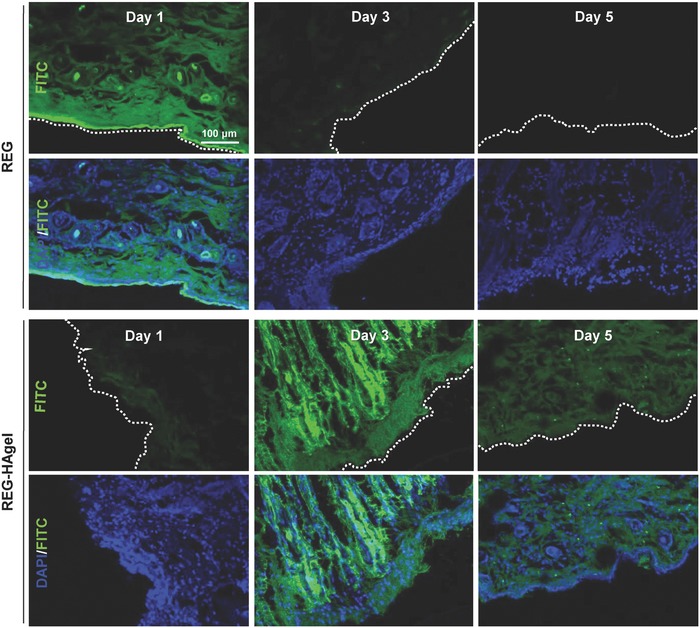
Dermal penetration of REG peptides. Fluorescence images of wound bed treated with FITC‐labeled REG and FITC‐labeled REG‐HAgel.

Prior to assessing wound healing effects of REG‐HAgels in vivo, the sustained peptide‐release properties of REG‐HAgels were further confirmed by histological assessment of dermal absorption and penetration of FITC‐labeled REG (**Figure** **5**). There was no significant difference in tissue penetration depth of REG with or without hydrogel formulation. When the REG solutions were simply applied onto the open wound, the strong fluorescence signal was observed throughout the epidermis on the first day of the treatment. Its fluorescence rapidly diminished 3 days after the treatment and appeared to be negligible after 5 days, indicating short retention time and fast diffusion rate of free peptides. In the case of REG‐HAgels, however, a small fraction of REG was released from hydrogels on day 1, which was mainly dependent on simple diffusion of the peptides. Then a significant amount of released peptide was observed on day 3. The sudden fast peptide release on day 3 might be attributed to the accelerated breakdown of HAgels by increased hyaluronidase in early wound healing.^21^ Many reports consistently demonstrate that degradation of HA hydrogels in vitro or in vivo strongly promotes the release of the payloads due to the increased mesh size of the HA hydrogels.^22^ The enhanced fluorescence signal of REG was gradually decreased over time. This result suggested that the HAgel‐based topical formulations allowed sustained delivery of the REG peptide at the wound site, leading to longer tissue residence time of the peptides maintaining within the therapeutic range.

### 2.4 In Vivo Wound Healing Effects of REG‐HAgels

**Figure 6:**
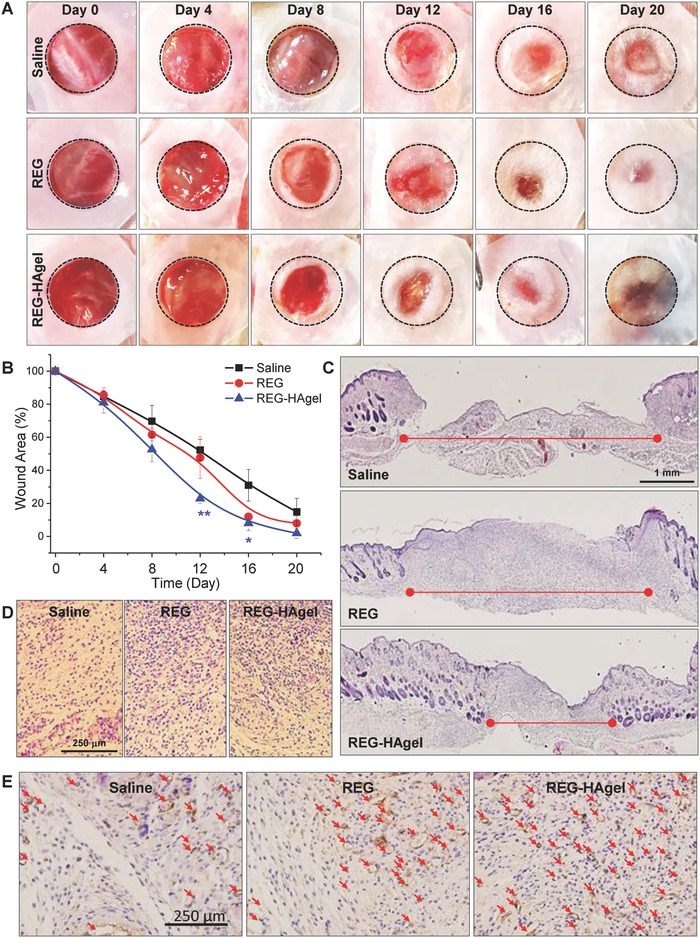
Therapeutic effect of REG‐HAgel in vivo. A) Macroview of mouse full‐thickness excisional wounds over 20 days of experiment and B) quantification of wound size. *n* = 5; *p < 0.05, **p < 0.01 versus saline‐treated wounds. C) Histological analysis of H&E stained sections of excisional wounds 8 days post‐treatment, and D) magnified images of H&E sections. E) Immunostaining against CD31.

A mouse cutaneous wound model was employed to evaluate the wound repair ability of REG in the topical formulation of REG‐HAgels (**Figure** **6**). The wounds treated with saline, REG, and REG‐HAgel were monitored photographically until the entire wound area was resurfaced (Figure 6A). When compared to the saline‐treated control group, the treatments of REG or HAgel alone showed slightly increased wound closure (Figure 6A; Figure S5, Supporting Information). With one‐time treatment of peptides, however, no statistically significant difference was detected between saline and REG groups. Considering that HAgels are able to sustainably release peptides and promote increased cell migration, potent synergistic effect between REG and HAgel was expected in wound repair. As shown in Figure 6B and Figure S5 (Supporting Information), REG‐HAgels markedly accelerated wound closure rate compared to both saline or REG. Differences of the wound size between the control and treatment groups started to appear on day 8. After 12 days of treatment, the wounds treated with REG‐HAgels exhibited 23% of open wound area, while 48% and 52% of wounds still remained open for saline and REG treated groups, respectively. As a result, the wounds treated with REG‐HAgels exhibited the most pronounced wound healing efficiency with complete wound closure occurring at day 16. The microscopic examination of hematoxylin–eosin (H&E) stained tissue sections also showed enhanced re‐epithelialization in REG‐HAgel treated groups compared to saline and REG treated groups (Figure 6C). Although the REG treatment showed a slight improvement in wound re‐epithelialization over the saline treated wounds, the REG‐HAgel formulation promoted more rapid re‐epithelialization resulting in strongly accelerated wound healing. After 12 days of treatment, the epithelial gap was decreased from 4.73 ± 0.24 mm in saline treated wounds to 4.25 ± 0.11 and 2.90 ± 0.18 mm in REG and REG‐HAgel treated wounds, respectively. During the remodeling state, the REG‐HAgel treatment could also improve the quality of wound healing with greater density of epithelial cells and higher collagen deposition in the dermal layer (Figure 6D), mainly attributed to the REG‐induced cell migration. Additional immunohistochemical analysis with CD31 antibodies further revealed that the underlying dermis of REG‐HAgel treated wounds possessed a higher density of microvessels compared with those of saline and REG treated groups (Figure 6E). It has been known that low molecular weight HA stimulates proliferation and migration of endothelial cells and promotes angiogenesis.^23^ Therefore, the REG‐HAgel wound dressing formulation may have facilitated faster wound repair by enzymatically cleaving HAgels into small HA fragments associated with enhanced angiogenesis. HA‐induced angiogenesis was also observed from the optical images of subcutaneous blank HAgel implants in Balb/c mice (Figure S6, Supporting Information). On day 5 of injection, the HAgel implants exhibited distinct vascular dilation and increased capillarization around the gel plug. The numerical and length density of microvessels increased with decreasing the size of HAgel over time, revealing that the HA fragments could play a key role in the angiogenesis acceleration at the wound site.

### 2.5 Activation of Downstream Signaling Pathways of FAK in Cell Migration In Vivo

**Figure 7:**
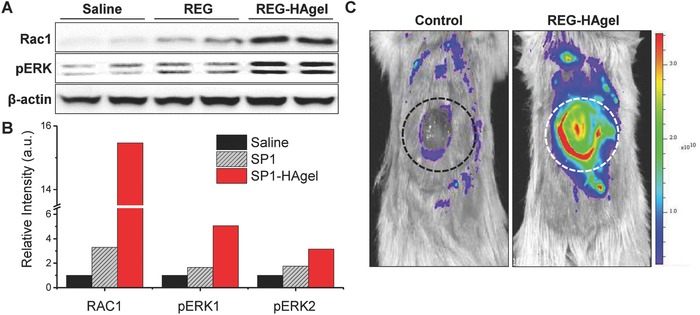
FAK downstream signals affected by REG‐HAgel treatment in vivo. A) Western blot analysis of Rac1, pERK1/2, and β‐actin with treatment of saline, REG, and REG‐HAgel. B) Quantification of protein expression levels. C) Fluorescence image of MMP2 activity surrounding the wound bed using MMP2‐activatable probe.

In order to investigate the molecular signaling pathways mediating the effects of REG‐HAgels on cell migration in wound healing in vivo, gene expression analysis on FAK‐dependent migratory signaling was performed on day 5 wound beds (**Figure** **7**). As used for in vitro experiments, the protein levels of Rac1 and pERK1/2 were assessed with western blot analysis and the expression levels of MMP2 were visualized using our MMP2‐specific molecular imaging probes. As shown in Figure 7A,B, the expression of Rac1 and pERK1/2 increased for both REG and REG‐HAgel groups compared to saline groups. In the REG‐HAgel treated wounds compared to the REG treated wounds, the protein levels of Rac1 and pERK1/2 dramatically increased by 15‐fold and fivefold, respectively. This result supported that sustainably delivering physiologically active REG peptides could successfully activate FAK‐dependent cell migratory signaling pathways. To visualize MMP2 activity at the wound site in real time, the MMP2 probe solution was applied directly to the wound surface overnight. The fluorescence images at the wound site were acquired with IVIS II Lumina (Figure 7C). The real time fluorescence imaging of wounds revealed that the REG‐HAgel treatment up‐regulated MMP2 production by activating Rac1 and pERK1/2, the key downstream effectors of FAK signaling pathways. With the strongest fluorescence signal detected at the edge of wound, the entire wound area indicated by the dotted circle also exhibited distinct fluorescence intensity, signifying the presence of MMP2 protein on the periwound skin. This result suggests that the subsequent ECM degradation mediated by activated MMPs allows for invasion of surrounding cells, such as dermal fibroblasts, epidermal keratinocytes, and endothelial cells, into the wound area.

## 3 Conclusion

In this study, we have created an advanced peptide‐grafted hydrogel dressing based on biocompatible porous HA hydrogels cross‐linked via copper‐free click chemistry. A novel synthetic wound healing peptide REG was successfully loaded into HA hydrogels. Due to the porous structure of HAgel and its biodegradability, the REG‐HAgel dressing formulation could sustainably release physiological active REG peptides on the wound surface, resulting in enhanced and prolonged therapeutic ability. The REG peptides released from HAgels strongly activated promigratory signals of FAK, such as Rac1 and pERK1/2, leading to increased MMP expressions during wound repair. Unlike the free REG treatment that simply modulates MMP‐dependent cell migration, the REG peptide‐embedded HAgel also acted synergistically to improve wound healing not only by regulating cell migration, but also by promoting angiogenesis. Consequently, the REG‐HAgel formulation enhanced both the rate and quality of wound healing with rapid re‐epithelialization, higher collagen deposition, and more microvascular formation. Our peptide‐grafted HAgel delivery system offers a promising new wound dressing platform for advanced wound care management by applying to a variety of therapeutic peptides.

## 4 Experimental Section

*Materials*: The effective peptide REG (REGRT, AES16‐2M) was selected and modified from the sequence of functional region of Erdr1 and the REG peptides were synthesized and supplied by Peptron Inc (Daejeon, Korea). BHQ‐3 was purchased from Biosearch Technologies (Novato, CA, USA). Cy5.5 succinimide ester was obtained from Amersham Bioscience (NJ, USA). Dimethylformamide (DMF), NHS, EDC, dimethylsulfoxide (DMSO), dimethylsulfoxide‐d6 (D, 99.9%), and deuterium oxide (D_2_O, D, 99.9%) were supplied by Sigma‐Aldrich Co (St. Louis, MO, USA). Sodium hyaluronate and 4‐Arm PEG‐azide (*M*_w_ 2 kDa) were purchased from Lifecore Biomedical LLC (Chaska, MN, USA) and Creative PEGWorks (Chapel Hill, NC, USA), respectively. Dibenzocyclooctyl‐PEG‐amine (DBCO‐PEG_4_‐NH_2_) was provided by Click Chemistry Tools (Scottsdale, AZ, USA). All antibodies were supplied by Cell Signaling (Danvers, MA, USA). All reagents and materials were obtained from Thermo Fisher Scientific (Pittsburgh, PA, USA) unless otherwise noted.

*Cell Culture*: nHDFs were obtained from the American Type Culture Collection (ATCC, Rochkville, USA). Cells were cultured according to the standard mammalian culture protocols and with sterile techniques. nHDF cells were cultured in Dulbecco's modified Eagle's medium (DMEM) supplemented with 10% fetal bovine serum and 1% antibiotic‐antimycotic. When the cells were ≈70–80% confluent, 0.5% trypsin‐ethylenediaminetetraacetic acid (EDTA) was used to passage the cells. Culture products listed above were purchased from Welgene Inc (Daegu, Korea).

*Western Blot Analysis*: Cells were plated in 90 mm cell culture dishes at a density of 2 x 10^5^ cells per dish. After 2 h of starvation, the cells were treated with the REG peptides, and lysed with radioimmunoprecipitation assay (RIPA) lysis buffer supplemented with 1% protease inhibitor. Protein content in the supernatants of lysates was quantified by bicinchoninic acid (BCA) assay and mixed with sodiumdodecyl sulfate (SDS) gel‐loading buffer. The samples corresponding to 40 µg of protein were separated by 12% SDS‐polyacrylamide gel electrophoresis and transferred onto polyvinylidene fluride membrane. Membranes were blocked with blocking buffer (5% skim milk in tris‐buffered saline containing tween 20 (TBST) solution, pH 7.4, 20 × 10^−3^ m Tris, 150 × 10^−3^ m NaCl, 0.05% Tween 20) for 1 h at room temperature, and incubated with primary antibodies for Rac1, pERK1/2, and β‐actin overnight at 4 °C. After incubating with primary antibodies, membranes were further incubated with secondary IgG‐HRP antibody for 1 h at room temperature. The membranes were briefly incubated with SuperSignal West Femto Maximum Sensitivity Substrate to develop the bands.

*Preparation of MMP2 Probe*: The MMP2‐enzyme activable peptide (MMP2 probe, Cy5.5‐Gly‐Pro‐Leu‐Gly‐Val‐Arg‐Gly‐Lys(BHQ3)‐Gly‐Gly‐OH) was synthesized with standard solid‐phase Fmoc peptide chemistry.^15^ To selectively conjugate BHQ‐3 to the primary amine of lysine, 2% hydrazine in DMF was added to remove Dde protecting group from Fmoc‐Gly‐Pro‐Leu‐Gly‐Val‐Arg(Pbf)‐Gly‐Lys(Dde)‐Gly‐Gly‐Resine. Subsequently, BHQ‐3 succinimide ester in anhydrous DMF was added to the peptide resin and reacted overnight. Fmoc group was removed and Cy5.5 succinimide ester was conjugated to the N‐terminal glycine of the peptide resin in a similar manner. The MMP2 peptide probe was removed from the resin with trifuoroactic acid and purified by reversed‐phase HPLC (Agilent Tech., CA, US).

*Visualization of In Vitro MMP2 Activity*: Cells were seeded in 35 mm glass bottom dishes at a density of 5 × 10^4^ cells per dish. Cells were incubated at 37 °C and 5% CO_2_ overnight. After 2 h of starvation, cells were treated with 100 ng of REG peptide loaded into 200 µL HAgel for 15 h prior to treating MMP2 probe. After carefully removing the hydrogel, the medium was replaced with fresh serum‐free culture medium, and the cells were incubated for 24 h to allow expression of MMP2. The cells were then incubated with MMP2 probe at a concentration of 10 µg mL^−1^ for 2 h. For fluorescence imaging, the cells were fixed with 4% paraformaldehyde for 10 min and counterstained with 4′,6‐diamidino‐2‐phenylindole (DAPI). The fixed cells were observed with Olympus fluorescence microscope IX81 (Tokyo, Japan).

*Migration Assay In Vitro*: Cells were seeded in 6‐well tissue culture at a density of 2 × 10^5^ cells per well. After starvation, cells were treated with the REG peptide, and the cell layers were scored with sterile 1 mL pipette tip from top to bottom. After 24 h of incubation, the cells were carefully washed with saline before taking microscopic images. Scoring of cells was visualized under Olympus CK40 culture microscope (Tokyo, Japan). All assays were carried out in quadruplicates.

*Preparation of HAgel*: One gram of sodium HA was dissolved in 200 mL of 1:1 (v/v) DMSO/deionized water followed by slowly adding 14.1 mg of NHS and 23.4 mg of EDC to activate the carboxylic groups. Concurrently, 43 mg of DBCO‐PEG_4_‐amine was dissolved in 1 mL DMSO. The DBCO‐PEG_4_‐amine solution was slowly added to the HA solution and vigorously stirred overnight at room temperature to synthesize DBCO‐PEG_4_‐HA (DBCO‐HA). The DBCO‐HA solution was purified by dialysis against methanol and deionized water for three days using dialysis tube with molecular weight cut‐off (MWCO) 12 kDa (Spectrum, Rancho Dominiquez, CA). The purified solution was lyophilized and redissolved in saline at desired concentrations. Unless stated otherwise, concentration of 20 mg mL^−1^ was used for in vitro and in vivo experiments. The final product was confirmed by ^1^H NMR. Prior to performing experiments with HAgels, the DBCO‐HA solution was cross‐linked with 4‐arm PEG‐azide cross‐linker by simply vortexing the mixture and incubating it at 37 °C until a defined gel was formed. To confirm structural properties of HAgel, ATR FT‐IR spectra of HA, HA‐DBCO, 4‐arm‐PEG‐azide, and HAgel were observed with 16 scans in the range of 4000–700 cm^−1^ by FT‐IR spectrometer (Nicolet iS10, Thermo Fisher, USA).

*Analysis of HAgel Properties*: ^1^H NMR (UnityPlus300, Varian, CA, USA) was used to analyze chemical compositions of the synthesized DBCO‐HA derivatives. HA, DBCO‐PEG_4_‐amine, and DBCO‐HA were dissolved in 1:1 (v/v) D_2_O/deuterated DMSO at a concentration of 10 mg mL^−1^, and the spectra were observed at 300 MHz. Rheology of HAgel was analyzed using dynamic mechanical analyzer (ELF3200, Endura TEC, Minnetonka, MN, USA). The storage modulus (*G*′) and loss modulus (*G*″) of each sample were measured at a stress of 25 Pa at room temperature. To analyze the swelling ratio, lyophilized hydrogels with known weights were immersed in saline and kept at 37 °C until the swelling reached equilibrium. At each time points, swollen hydrogels were weighed, and equilibirum swelling ratio (ESR) was determined through following equation

(1) $$\mathrm{ESR}=\left(W_{s}-W_{d}\right)/W_{d}$$

where *W*_s_ represents the weight of swollen hydrogel and *W*_d_ represents the weight of dried hydrogel. SEM (JSM‐6330F, Peabody, MA, USA) was used to visualize the cross‐sectional morphology of HAgels with different concenetrations of 4‐arm PEG azide. The lyophilized samples were cut to expose the cross‐sectional area and placed on a carbon tape. A thin layer of platinum was coated on the surface of HAgels to enhance visualization.

*Cytotoxicity Assay*: To assess in vitro biocompatibility of Hagels, viability of the cells encapsulated in HAgel was examined via live/dead assay. Cells were suspended in 100 µL culture medium and mixed with the DBCO‐HA solution at a density of 1 × 10^7^ cells per 500 µL soltuion via lightly vortexing the mixture. The DBCO‐HA solution containing nHDF was cross‐linked with 0.5 × 10^−3^ m 4‐arm PEG azide and placed on a 6‐well tissue culture plate at 37 °C until a defined hydrogel was formed. The cell‐encapsulated HAgels were incubated in fresh culture medium for 3 and 5 days. The gels were washed with saline and incubated with Cellstain Double Staining Kit (Sigma‐Aldrich Co., St. Louis, MO, USA). The stained cell‐encapsulated HAgels were transferred to glass microscopy slides for visualization. Cover glasses were carefully pressed against the slides to flatten out the gels. The cells were visualized under a laser scanning confocal microscopy (LSM 510 Meta, Carl Zeiss Inc., Thornwood, NY).

*REG Release Profile from HAgel*: FITC‐labeled REG was used to quantify the amount of REG released from HAgel. One hundred nanograms of REG was mixed with 200 µL DBCO‐HA solution and cross‐linked with 4‐arm PEG azide. The peptide encapsulated HAgels were briefly washed with saline to remove excess REG on the surface of HAgel. Subsequently, HAgels were sumberged in a fresh 1 mL saline and incubated at 37 °C. The buffer, containing released peptides, was collected at each indicated time point and stored at 4 °C until the last day of collection. The samples were analyzed at 519 nm using VERSAmaxTM microplate reader (Sunnyvale, CA, USA).

*Mass Spectral Analysis of REG*: The REG peptide released from REG‐HAgels and the synthetic REG peptide were desalted by Sep‐Pack tC18 solid‐phase extraction cartridges (Waters Corporation, Milford, MA) and dried in vacuo. Each dried peptide was resuspended using 20 µL of 0.4% acetic acid, and 10 µL of each peptide sample was injected onto a 1.5 cm, 100 µm i.d. trap column (ReproSil‐Pur C_18_ resin, 200 Å, 5 µm, Dr.Maisch GmbH, Ammerbuch‐Entringen, Germany) and separated on a 15 cm, 75 µm i.d. analytical column (ReproSil‐Pur C_18_ resin, 200 Å, 3 µm) using a 2D NanoLC pump from Eksigent Technologies (Dublin, CA, USA). The operating flow rate was 300 nL min^−1^ with the following gradient conditions: 0 min at 100% buffer A (100% water with 0.1% formic acid) and 0% buffer B (100% acetonitrile with 0.1% formic acid), 0–5 min at 0–6% buffer B, 5–30 min at 6–15% buffer B, 30–35 min at 15–70% buffer B, 35–45 min at 70% buffer B, 45–50 min at 70–2% buffer B, and 50–60 min at 2% buffer B. Data were collected on an LTQ‐Orbitrap XL mass spectrometer (Thermo Scientific, San Jose, CA, USA), using the Orbitrap mass analyzer with AGC targets of 1 × 10^6^ for MS (7500 resolving power at *m*/*z* 400) and scan range of 250–350 *m*/*z*. The spray voltage was set to 2.5 kV, and the temperature of the heated capillary was held at 250 °C.

*Cellular Uptake of REG Released from HAgel*: To visualize cellular uptake of REG released from HAgel, 100 ng of FITC‐labeled REG was encapsulated in 200 µL hydrogel. Cells were seeded at a density of 1 × 10^5^ cells per 35 mm glass bottom dish. After starvation, REG‐HAgels were located on a side of the dish to prevent disruption of cell monolayer. The cells were incubated with REG‐HAgel overnight, and the dishes were gently shaken several times so that REG is evenly distributed. Fluorescence imaging was performed as described above.

*Full‐Thickness Animal Excisional Wound Model*:^24^ Balb/c mice (male, 5 weeks) were purchased from Nara Biotech (Seoul, Korea). All animal experiments were performed in compliance with the International Guide for the Care and Use of Laboratory Animals. All experimental procedures were examined and approved by Korea Institute of Science and Technology (IACUC2017‐086). To produce excisional rodent wound model, mice were anesthetized, and the hair was removed from the dorsal surface. The skin surface was disinfected with 10% (w/v) povidone–iodine and 70% (v/v) ethanol. Two identical full‐thickness excisional wounds were made with 8 mm Integra Miltex disposable biopsy punch (York, PA, USA) on the dorsum of each mouse, and 0.5 mm thick silicon splinting ring was secured around the wound with instant‐bonding adhesive (Scotch Super Glue liquid; 3M, St Paul, MN, USA) to prevent skin contraction. After topically treating the wounds with saline, 50 µg REG peptide solution, or 50 µg REG peptide in 100 µL HAgels, wounds were photographed using digital camera. Wound sites were completely covered with sterile Tegaderm (3M Health Care, St. Paul, MN, USA) dressing and secured with self‐adhering elastic bandage (Coban; Johnson & Johnson, Arlington, TX, USA).

*Histology*: To assess the dermal absorption and penetration of REG through the wounded skin, FITC‐labeled REG was used for fluorescence imaging. After creating the wounds as described above, wounds were treated with 200 µg REG or 200 µg REG peptide in 100 µL HAgels, and animals were sacrifced 1, 3, and 5 days after the treatment. Wound bed was excised from the dorsal surface and fixed with 20% followed by 40% (w/v) sucrose solution for 2 h each. The fixed samples were embedded in O.C.T compound and frozen at −80 °C for cryostat sectioning. The tissue sections (10 µm in thickness) were counter‐stained with DAPI solution, and FITC‐labeled REG was visulized under confocal laser scanning microscopy. For H&E staining and immunohistochemistry (IHC), the tissue samples were fixed in 10% phosphate‐buffered formalin overnight, dehydrated, and embedded in paraffin. The paraffin‐embedded tissue blocks were sectioned into 6 µm slices and deparaffinizaed and hydrated in a series of xylene and ethanol solutions. The tissue slides were either stained with hematoxylin–eosin or prepared with Histostain‐Plus IHC Kit (Invitrogen, Carlsbad, CA, USA). For IHC, monoclonal mouse anti‐CD31 antibody (dilution 1:200) was used, and the tissue samples were prepared according to the manufacturer's protocol.

*Visualization of In Vivo MMP2 Activity*: Wounds were treated with saline, 50 µg of REG, 100 µL HAgel, or 50 µg REG in 100 µL HAgel. Five days after the treatment, 1 mg dark‐quenched fluorogenic peptide was dispersed in saline buffer and applied topically onto the wounds. Subsequently, the wounds were sealed with Tegaderm dressing and Coban elastic bandages. After 24 h, the wound bed was washed with saline to remove the remnants of MMP2 probe, then fluorescence imaging was performed using IVIS II Lumina. (Caliper LifeSciences, Hopkinton, MA).

*Statistical Analysis*: All data were analyzed using Origin pro 8 software package (OriginLab Corp, MA, USA). All groups were compared with student *t*‐test or one‐way ANOVA, and a value of *p* < 0.05 was considered statistically significant.

## Conflict of Interest

The authors declare no conflict of interest.

## Supporting information

Supplementary
