# Supplementary material for: Development of Biocompatible HA Hydrogels Embedded with a New Synthetic Peptide Promoting Cellular Migration for Advanced Wound Care Management
Source: Adv Sci (Weinh). 2018 Sep 21;5(11):1800852. doi: 10.1002/advs.201800852 (PMC6247053; doi:10.1002/advs.201800852)
Supplement: Supplementary file 1 — Supplementary [file ADVS-5-1800852-s001.pdf]

Copyright WILEY-VCH Verlag GmbH & Co. KGaA, 69469 Weinheim, Germany, 2018.

ADVANCED SCIENCE Open Access

Supporting Information

for *Adv. Sci.,* DOI: 10.1002/advs.201800852

Development of Biocompatible HA Hydrogels Embedded with a New Synthetic Peptide Promoting Cellular Migration for Advanced Wound Care Management

Sun Young Wang, Hyosuk Kim, Gijung Kwak, Hong Yeol Yoon, Sung Duk Jo, Ji Eun Lee, Daeho Cho, Ick Chan Kwon, and Sun Hwa Kim**

Copyright WILEY-VCH Verlag GmbH & Co. KGaA, 69469 Weinheim, Germany, 2016.

Supporting Information

Development of Biocompatible HA Hydrogels Embedded with a New Synthetic Peptide Promoting Cellular Migration for Advanced Wound Care Management

Sun Young Wang, Hyosuk Kim, Gijung Kwak, Hong Yeol Yoon, Sung Duk Jo, Ji Eun Lee, Daeho Cho, Ick Chan Kwon, and Sun Hwa Kim**

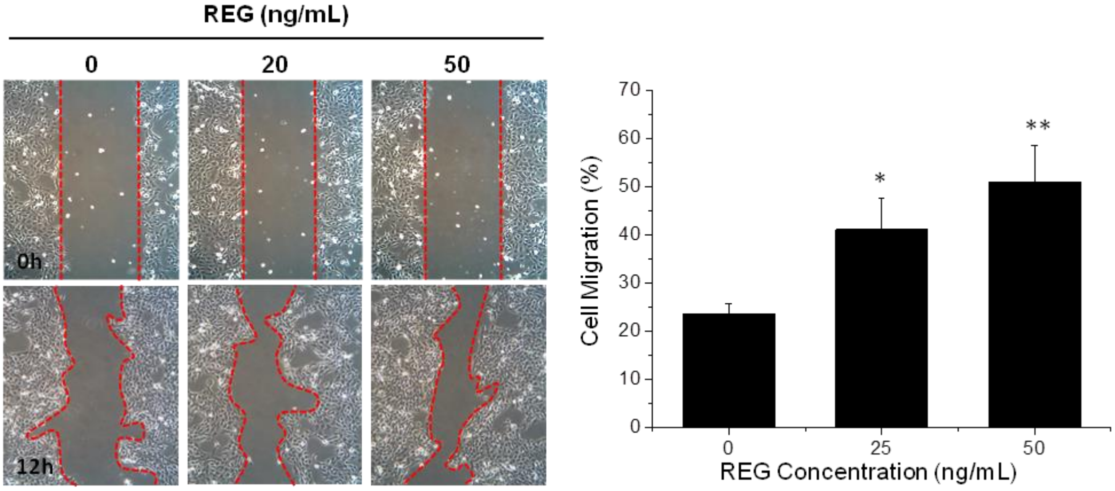

Figure S1. Wound scratch assay with HaCat keratinocytes and quantification of migrated cells. *n* = 4, * *p*<0.05, ** *p*<0.01 *vs* saline-treated cells.

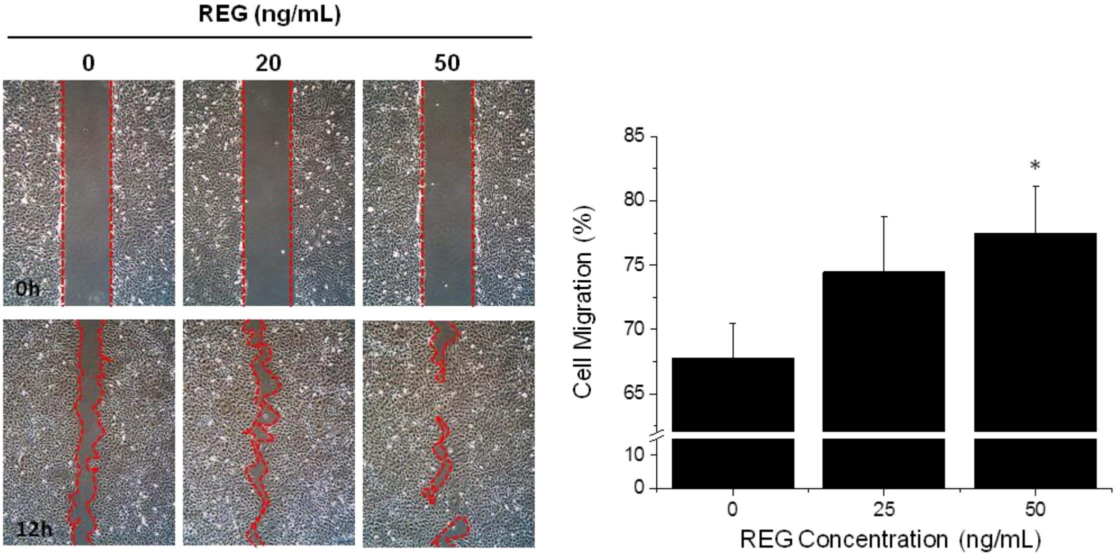

Figure S2. Wound scratch assay with SVEC4 endothelial cells and quantification of migrated cells. $n = 4$, * $p<0.05$ *vs* saline-treated cells.

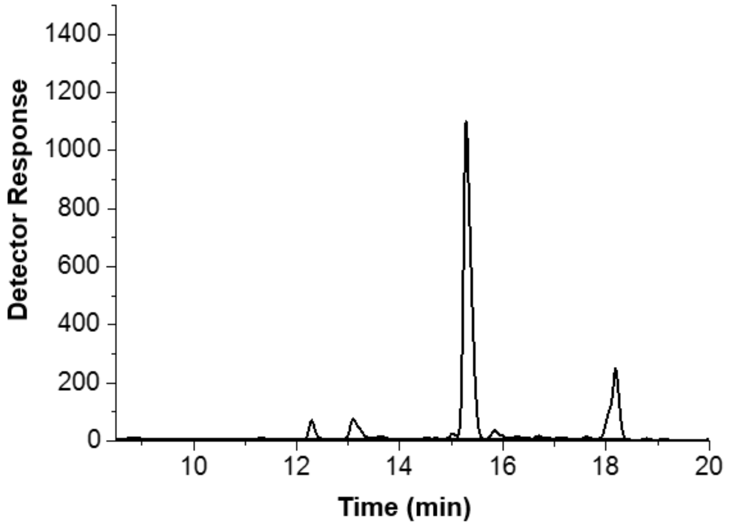

Figure S3. HPLC analysis of MMP2-activatable probe. The peak at 15.3 min retention time indicates a successful conjugation of MMP2 peptide probe.

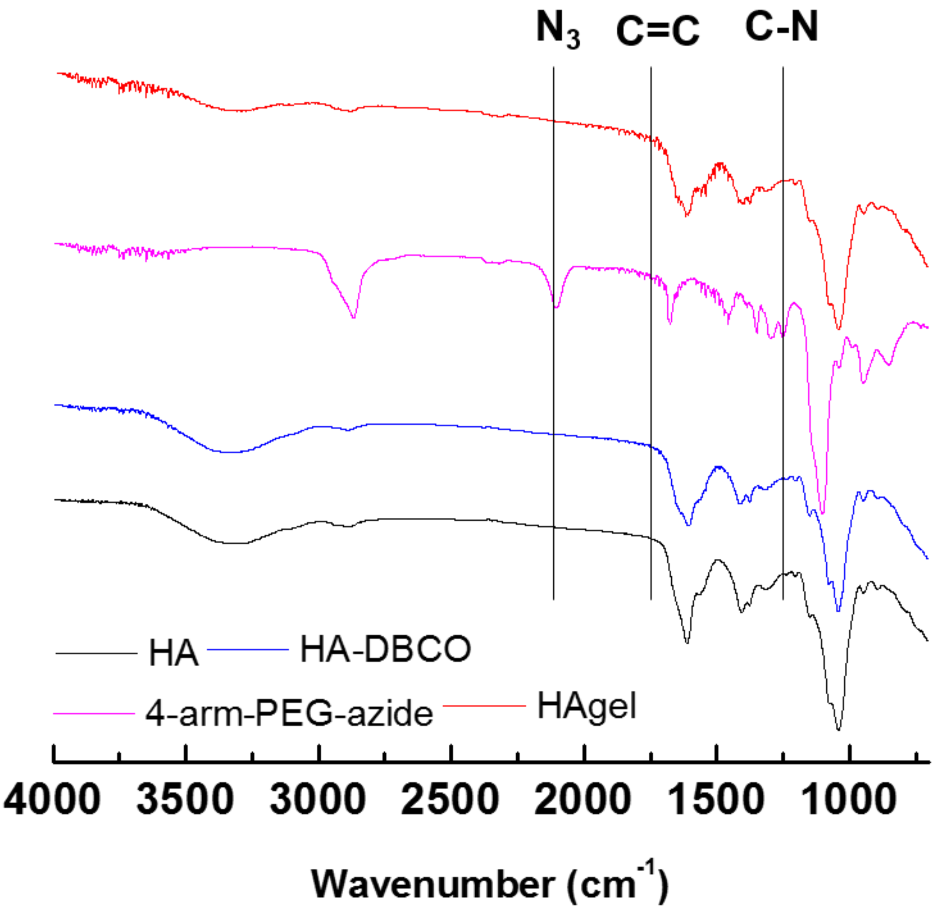

Figure S4. ATR FT-IR spectra of HA, HA-DBCO, 4-arm-PEG-azide and HAgel in the range of 4000 to 700 cm^{-1}.

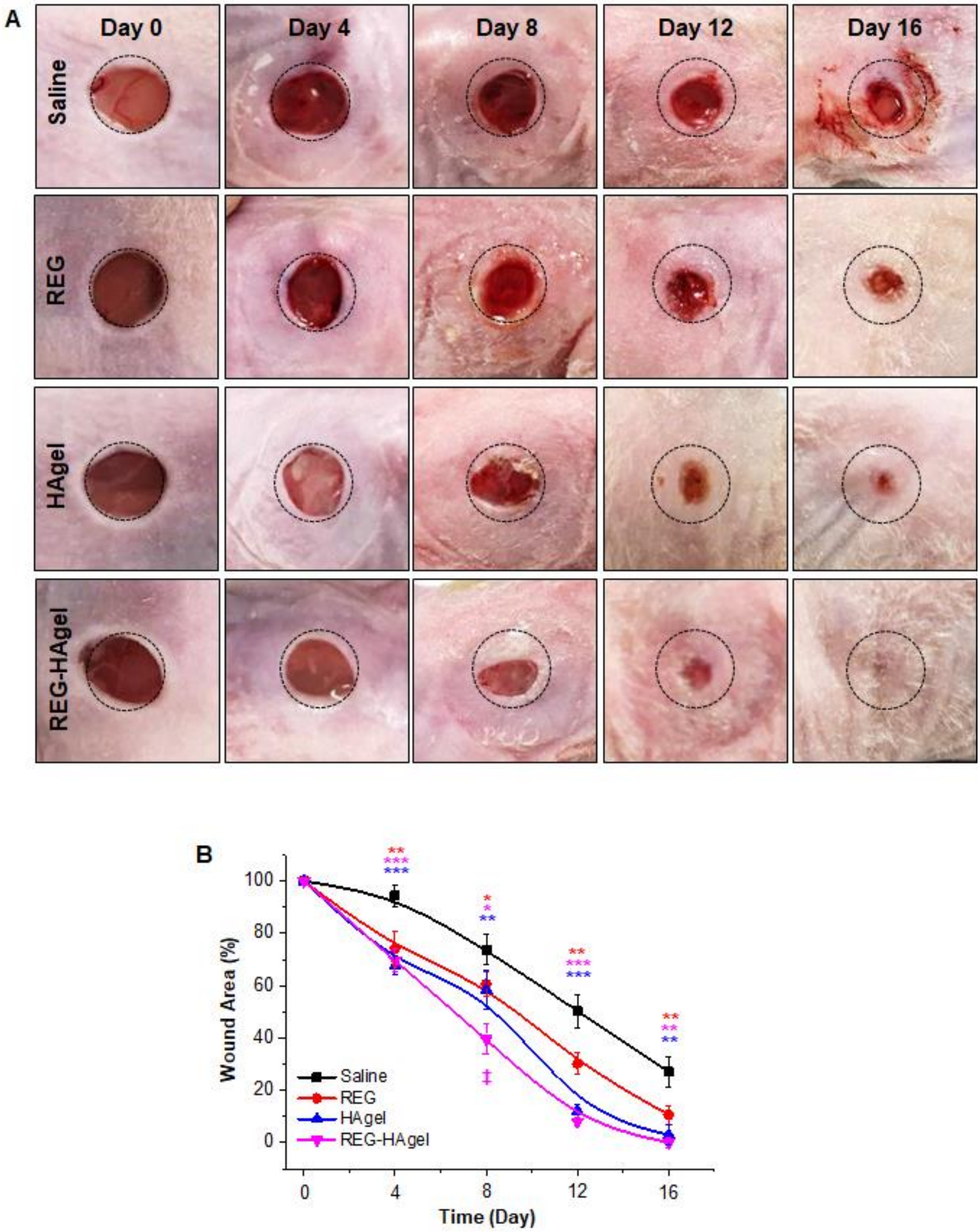

Figure S5. Therapeutic effect of REG, HAgel and REG-HAgel in Balb/c nude mouse. A) Representative photographic images of 6 mm wide full-thickness wounds over 16 days of experiment, and B) quantification of wound sizes. n = 3; * p<0.05, ** p<0.01, *** p<0.005 *vs* Saline. ‡ p<0.01 *vs* HAgel.

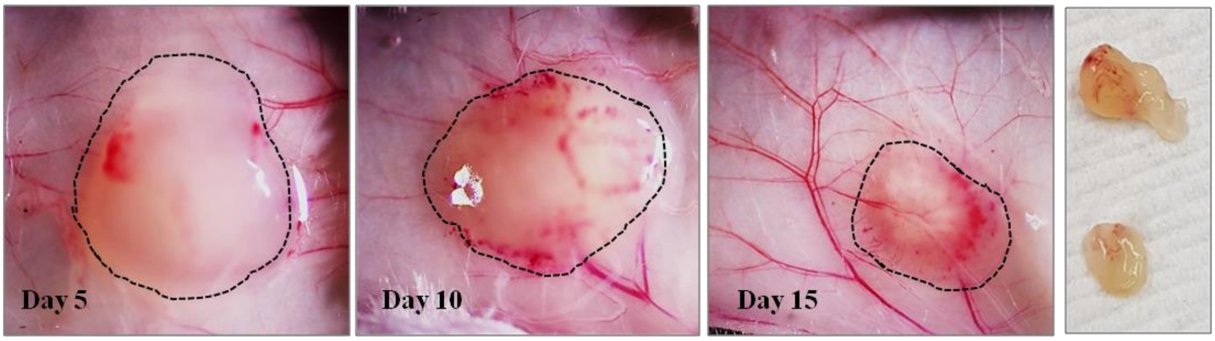

Figure S6. Optical images of subcutaneously implanted HAgel observed over 15 days.
